# ﻿A new species of the genus *Leptobrachella* (Amphibia, Anura, Megophryidae) from Dayaoshan National Nature Reserve, Guangxi, China

**DOI:** 10.3897/zookeys.1219.121027

**Published:** 2024-11-27

**Authors:** Gui-Dong Yu, Kun Qin, Tao Meng, Peng Li, Wan-Xiao Peng, Wei-Cai Chen

**Affiliations:** 1 Guangxi Forest Inventory and Planning Institute, Nanning 530001, China; 2 Guangxi Dayaoshan Forest Ecosystem Research Station, Jinxiu 545700, China; 3 Laibin Jinxiu Dayaoshan Forest Ecosystem Observation and Research Station of Guangxi, Jinxiu 545700, China; 4 Key Laboratory of Environment Change and Resources Use in Beibu Gulf Ministry of Education, Nanning Normal University, Nanning 530001, China; 5 Guangxi Key Laboratory of Earth Surface Processes and Intelligent Simulation, Nanning Normal University, Nanning 530001, China

**Keywords:** Bioacoustics, cryptic diversity, morphology, phylogeny, taxonomy

## Abstract

A new species of the Asian leaf litter toad genus *Leptobrachella*, *L.dayaoshanensis***sp. nov.**, is described based on phylogenetic analysis, morphological characters, and bioacoustic data. This species occurs in the Dayaoshan National Nature Reserve located in Jinxiu County, Guangxi, China. Phylogenetic analysis indicates that this new species is closely related to *L.verrucosa*, as demonstrated by phylogenetic trees. The new species can be distinguished from its congeners by a combination of the following characters: (1) medium size (mean snout–vent length (SVL) of 27.9 ± 0.7 mm, range 26.6–28.9 mm in males; 34.4 mm in female); (2) rough dorsal surface featuring small, raised tubercles and ridges; (3) flanks adorned with irregular black spots and creamy white glands; (4) creamy white ventral surface with sparse light-brown spots and irregular tiny textures; (5) brown throat and chest; (6) rudimentary toe webbing; (7) wide lateral fringes on toes; (8) distinct continuous ventrolateral glandular line; (9) tibiotarsal articulation reaching the midpoint of eye when the leg is extended forward; (10) heels that do not meet when thighs are appressed at right angles to body; (11) bicolored iris, with the upper half being copper and gradually transitioning to silver in the lower half; and (12) advertisement calls consisting of two model types, with dominant frequencies of 4.2–6.8 kHz at 21.0 °C. The new species has a breeding season that occurs from March to April and is found in evergreen forests at elevations between 1,000 and 1,600 m.

## ﻿Introduction

*Leptobrachellaliui* (Fei & Ye, 1990) is known to have a wide distribution in eastern and southern China (AmphibiaChina 2024; Frost 2024). In 1931, Pope collected 13 specimens from Chong’an (now Wuyishan City), Fujian, China, and initially classified them as *Megophryspelodytoides*. Later, they were transferred to the genus *Leptolalax* Dubois, 1980, and then to *Leptobrachella* Smith, 1925 (Chen et al. 2018). However, Pope (1931) also noted that the Fujian specimens differed from the holotype (Boulenger 1893; Smith 1930), mainly in the number of law teeth of tadpoles (4 lines vs 5–6 lines). In 1950, Liu (1950) examined the Fujian specimens collected by Pope and argued that they belonged to an unknown species in the genus *Megophrys* (Liu 1950; Fei et al. 1992). In 1990, Fei et al. (1990) classified the Fujian specimens as a new species, *Leptobrachellaliui*, and subsequently redescribed them (Fei et al. 1992). They also assigned specimens from Dayaoshan and Longsheng, Guangxi, Leishan, Guizhou, Yizhang, Hunan, and Zhejiang provinces to *L.liui*, despite some morphologic variations. Later reports indicated that *L.liui* was widely distributed in Fujian, Zhejiang, Jiangxi, Hunan, Guangdong, and Guangxi provinces (Fei et al. 2012; Mo et al. 2014; Shen 2014; Fei and Ye 2016; Chen et al. 2018). Based on molecular data, Chen et al. (2018) initially discovered that specimens of *L.liui* from various geographic populations can be categorized into three distinct lineages, forming the *L.liui* species complex. The first lineage, found in the Mangshan Nature Reserve, Yizhang County, Hunan, China, was identified as *L.mangshanensis*, representing the first cryptic species (Chen et al. 2018; Hou et al. 2018). The second lineage, discovered in the Dawuling Forest Station, Maoming City, Guangdong, China, was named *L.yunkaiensis*, corresponding to the second cryptic species (Chen et al. 2018; Wang et al. 2018). The third lineage was identified as *L.liui*, representing the sensu stricto *L.liui*. Interestingly, within the second lineage, a specific sequence (GenBank no. EF544238) from Dayaoshan, Jinxiu County, Guangxi, China, exhibited a significantly shorter evolutionary branch and a relatively larger genetic divergence compared to *L.yunkaiensis*, indicating the presence of an unknown species within *Leptobrachella*. Recently, we collected 16 specimens from Dayaoshan, Jinxiu County, Guangxi. Through thorough examination of morphology, phylogeny, and bioacoustics, we observed distinct differences between these specimens, previously classified as *L.liui*, and both the sensu stricto *L.liui* from the type locality and *L.yunkaiensis*. Therefore, we propose the classification of these specimens as a new species within the genus *Leptobrachella*.

## ﻿Material and methods

### ﻿Sampling

**Figure 1.**
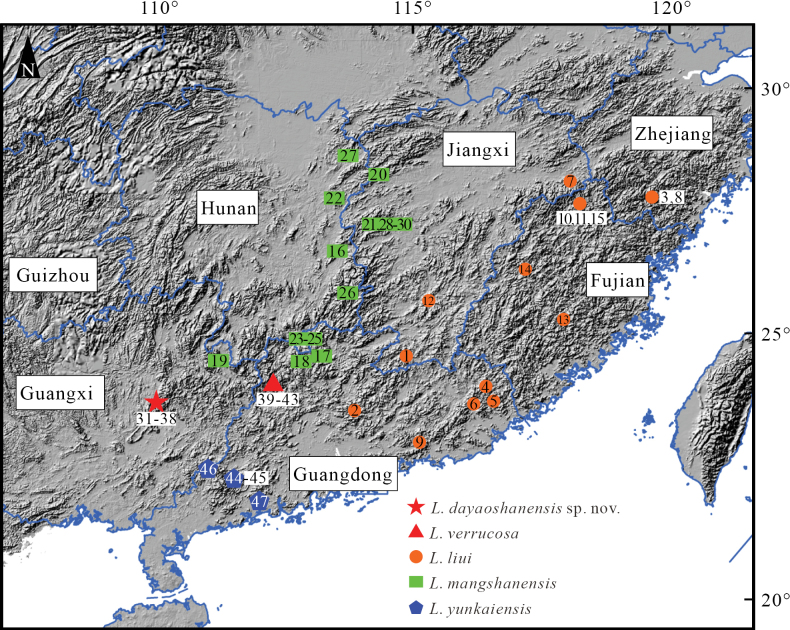
Localities of the new species and its sister taxa.

Sixteen specimens were collected from Dayaoshan (**DYS**), Jinxiu County, Guangxi, China (Fig. 1). All specimens were initially fixed in 10% formalin for 48 h and subsequently transferred to 75% ethanol for permanent storage at Nanning Normal University (**NNU**). Prior to fixation, muscle tissues were extracted and stored in 100% ethanol for molecular analysis.

### ﻿Morphometrics and morphological comparisons

All specimens were measured following the methodology described in Rowley et al. (2016). Measurements were taken using a digital caliper with an accuracy of 0.1 mm. The following morphological characters were measured: snout–vent length (**SVL**), head length (**HL**) from the tip of the snout to the rear of the jaws, head width (**HW**) at the commissure of the jaws, snout length (**SNT**) from the tip of the snout to the anterior eye corner, diameter of the exposed portion of the eyeball (**ED**), interorbital distance (**IOD**), which is the shortest distance between the anterior corners of the orbits, horizontal diameter of the tympanum (**TD**), distance from the anterior edge of the tympanum to the posterior eye corner (**TED**), internarial space distance (**IN**), tibia length (**TIB**) with flexed hindlimb, forelimb length (**FLL**) from the elbow to the tip of the third finger, length of the foot and tarsus (**TFL**) from the tibiotarsal articulation to the distal end of toe IV, manus length (**ML**) from the tip of the third digit to the proximal edge of the inner palmar tubercle, hindlimb length (**HLL**) from the tip of the fourth toe to the vent, and the distance from the proximal edge of the femoral gland to the knee (**FG-knee**). Gender was determined through direct observation of calls in life or the presence of internal vocal sac openings.

Comparative morphological data were obtained from the references (Suppl. material 2: table S1) and museum specimens (Suppl. material 2: table S2). For morphological comparisons, we used 15 adult males from the new specimens to compare with their closely related species, including *Leptobrachellaliui* (type locality, male, *n* = 6), *L.mangshanensis* (type locality, male, *n* = 6), and *L.verrucosa* (holotype and paratypes, male, *n* = 5), based on the results of phylogenetic analyses. We performed Principal Component Analyses (PCA) and the Mann-Whitney *U* test using SPSS v. 20. Before analysis, the measurements were transformed as follows: we calculated the ratio of each morphometric character (except SVL itself) to SVL, and then log-transformed them for morphometric analyses.

### ﻿Molecular analyses

Genomic DNA was isolated from muscle tissue using tissue extraction kits (Tiangen Biotech Co. Ltd., Beijing, China). Mitochondrial fragments of the 16S gene were amplified and sequenced using the primers: 16Sar_L, 5′–CGCCTGTTTACCAAAAA CAT–3′; 16Sbr_H, 5′–CCGGTCTGAACTCAGATCACGT–3′) (Palumbi et al. 1991). Polymerase chain reaction (PCR) amplification was performed as described by Chen et al. (2021). The fragments were sequenced on an automated DNA sequencer (ABI Prism 3730, Applied Biosystems, USA). The newly obtained sequences were deposited in GenBank (accession numbers: PQ476281–PQ476287). Phylogenetic relationships were reconstructed using maximum-likelihood (ML) and Bayesian-inference (BI) analyses. The sequence information used in the molecular analysis is provided in Table 1. The best-fit nucleotide substitution models (GTR+F+I) were selected using ModelFinder v. 2.2.0 (Kalyaanamoorthy et al. 2017; Zhang et al. 2020) based on the Bayesian information criterion (BIC). ML analyses were performed in IQ-tree v. 2.2.2 (Nguyen et al. 2015) with 2000 ultrafast bootstrap replicates. BI was conducted using MrBayes v. 3.2 (Ronquist et al. 2012). Two independent runs with four Markov Chain Monte Carlo simulations were performed for 30 million iterations, and trees were sampled every 1,000^th^ generation. The first 25% of trees were discarded as burn-in. Uncorrected pairwise distances were calculated using Mega v. 7 with the default settings (Kumar et al. 2016).

**Table 1.** DNA sequences used in this study. ‘*’ represents type locality. ‘#’ means the sequences named *Leptobrachellaliui* from Chen et al. (2018).

ID	Species	Locality	Voucher no.	16S	Note^#^
1	*L.liui*	Jiulian Shan, Jiangxi, China	SYS a002105	MH055911	*L.liui*
2	*L.liui*	Nankunshan Nature Reserve, Guangdong, China	SYS a004497	MH055924	*L.liui*
3	*L.liui*	Dongkeng Town, Jingning County, Zhejiang, China	SYSa002732	MH605575	*L.liui*
4	*L.liui*	Tongguzhang, Guangdong, China	SYS a004733	MH055912	*L.liui*
5	*L.liui*	Jiaoshuikeng, Guangdong, China	SYS a003698	MH055913	*L.liui*
6	*L.liui*	BaiTang, Guangdong, China	KIZ018349	MH055914	*L.liui*
7	*L.liui*	Tongba Shan, Jiangxi, China	SYS a001702	KM014548	*L.liui*
8	*L.liui*	Dongkeng, Zhejiang, China	SYS a002733	MH055909	*L.liui*
9	*L.liui*	Gutian Nature Reserve, Guangdong, China	SYS a002650	MH055910	*L.liui*
10	*L.liui*	Wuyishan, Fujian, China *	SYS a001597	KM014547	*L.liui*
11	*L.liui*	Wuyishan, Fujian, China *	SYS a002478	MH605573	*L.liui*
12	*L.liui*	Huanggangshan, Jiangxi, China	SYS a001620	KM014549	*L.liui*
13	*L.liui*	Daiyunshan, Fujian, China	SYS a001736	KM014550	*L.liui*
14	*L.liui*	Longqishan, Fujian, China	SYS a002506	MH055907	*L.liui*
15	*L.liui*	Wuyishan, Fujian, China *	ZYCA907	MH055908	*L.liui*
16	*L.mangshanensis*	Longzha, Hunan, China	SYS a002539	MH055918	*L.liui*
17	*L.mangshanensis*	Tianjingshan National Forest Park, Guangdong, China	SYS a002805	MH055921	*L.liui*
18	*L.mangshanensis*	Nanling National Forest Park, Guangdong, China	SYS a002829	MH055920	*L.liui*
19	*L.mangshanensis*	Dadong Shan, Guangxi, China	SYS a002848	MH055922	*L.liui*
20	*L.mangshanensis*	Wugongshan, Jiangxi, China	SYS a004035	MH055916	*L.liui*
21	*L.mangshanensis*	Jinggangshan, Jiangxi, China	SYS a004051	MH055917	*L.liui*
22	*L.mangshanensis*	Yunyangshan, Hunnan, China	SYS a002540	MH055923	*L.liui*
23	*L.mangshanensis*	Mangshan, Hunan, China*	MSZTC201701	MG132196	*L.liui*
24	*L.mangshanensis*	Mangshan, Hunan, China*	MSZTC201702	MG132197	*L.liui*
25	*L.mangshanensis*	Mangshan, Hunan, China*	MSZTC201703	MG132198	*L.liui*
26	*L.mangshanensis*	Guidong, Hunan, China	SYS a002521	MH055919	*L.liui*
27	*L.mangshanensis*	Yangxi, Jiangxi, China	SYS a002578	MH055915	*L.liui*
28	*L.mangshanensis*	Mt. Jinggang, Jiangxi, China	SYSa004044	MH406906	*L.liui*
29	*L.mangshanensis*	Mt. Jinggang, Jiangxi, China	SYSa004045	MH406907	*L.liui*
30	*L.mangshanensis*	Mt. Jinggang, Jiangxi, China	SYSa004052	MH406908	*L.liui*
31	*L.dayaoshanensis* sp. nov.	Dayaoshan, Jinxiu County, Guangxi, China*	ZYC A100	EF544238	*L.liui*
32	*L.dayaoshanensis* sp. nov.	Dayaoshan, Jinxiu County, Guangxi, China*	NNU202103018	PQ476281	
33	*L.dayaoshanensis* sp. nov.	Dayaoshan, Jinxiu County, Guangxi, China*	NNU202103019	PQ476282	
34	*L.dayaoshanensis* sp. nov.	Dayaoshan, Jinxiu County, Guangxi, China*	NNU202103021	PQ476283	
35	*L.dayaoshanensis* sp. nov.	Dayaoshan, Jinxiu County, Guangxi, China*	NNU202103022	PQ476284	
36	*L.dayaoshanensis* sp. nov.	Dayaoshan, Jinxiu County, Guangxi, China*	NNU202103027	PQ476285	
37	*L.dayaoshanensis* sp. nov.	Dayaoshan, Jinxiu County, Guangxi, China*	NNU202103028	PQ476286	
38	*L.dayaoshanensis* sp. nov.	Dayaoshan, Jinxiu County, Guangxi, China*	NNU202103032	PQ476287	
39	*L.verrucosa*	Lianshan Bijiashan Nature Reserve, Guangdong, China	GEP a059	OP279589	
40	*L.verrucosa*	Lianshan Bijiashan Nature Reserve, Guangdong, China	GEP a060	OP279590	
41	*L.verrucosa*	Lianshan Bijiashan Nature Reserve, Guangdong, China	GEP a061	OP279591	
42	*L.verrucosa*	Lianshan Bijiashan Nature Reserve, Guangdong, China	GEP a062	OP279592	
43	*L.verrucosa*	Lianshan Bijiashan Nature Reserve, Guangdong, China	GEP a063	OP279593	
44	*L.yunkaiensis*	Dawushan, Guangdong, China*	SYS a004666	MH055933	*L.liui*
45	*L.yunkaiensis*	Dawushan, Guangdong, China*	SYS a004663	MH605584	
46	*L.yunkaiensis*	Lidong, Guangxi, China	KIZ018211	MH055931	*L.liui*
47	*L.yunkaiensis*	Ehuangzhang Nature Reserve, Guangdong, China	KIZ047782	MH055932	*L.liui*
48	*L.aerea*	Quang Binh, Vietnam	ZFMK 86362	JN848409	
49	*L.alpina*	Caiyanghe, Yunnan, China	KIZ049024	MH055867	
50	*L.applebyi*	Phong Dien Nature Reserve, Thua Thien-Hue, Vietnam	KIZ010701	MH055947	
51	*L.arayai*	Borneo, Malaysia*	AE100/S9	DQ642119	
52	*L.ardens*	Kon Ka Kinh National Park, Gia Lai, Vietnam*	ZMMU-NAP-06099	MH055949	
53	*L.aspera*	Huanglianshan Nature Reserve, Lyuchun, Yunnan, China*	SYS a007743	MW046199	
54	*L.baluensis*	Sabah, Borneo, Malaysia*	SP 21604	LC056792	
55	*L.bashaensis*	Basha Nature Reserve, Guizhou, China*	GIB196404	MW136295	
56	*L.bidoupensis*	Bidoup-Nui Ba National Park, Lam Dong, Vietnam*	ZMMU-A-4797-01454	MH055945	
57	*L.bijie*	Bijie City, Guizhou, China*	SYS a007313	MK414532	
58	*L.botsfordi*	Lao Cai, Vietnam*	AMS R 176540	MH055952	
59	*L.bourreti*	Mao’ershan, Guangxi, China	KIZ019389	MH055869	
60	*L.brevicrus*	Sarawak, Borneo, Malaysia*	ZMH A09365	KJ831302	
61	*L.chishuiensis*	Guizhou, China*	CIBCS20190518047	MT117053	
62	*L.crocea*	Thua Thien-Hue, Vietnam	ZMMU-NAP-02274	MH055955	
63	*L.damingshanensis*	Wuming County, Guangxi, China*	NNU202103281	MZ145229	
64	*L.dong*	Tongdao County, Hunan, China*	CIB SSC1757	OP764530	
65	*L.dorsospina*	Yushe Forest Park, Shuicheng, Guizhou, China*	SYS a004961	MW046194	
66	*L.dringi*	Borneo, Malaysia*	KUHE:55610	AB847553	
67	*L.eos*	Phongsaly, Laos*	MNHN 2004.0274	JN848452	
68	*L.feii*	Yunnan, China*	KIZ048894	MT302634	
69	*L.firthi*	Kon Tum, Vietnam*	AMS: R 176524	JQ739206	
70	*L.flaviglandulosa*	Xiaoqiaogou Nature Reserve, Yunnan, China*	KIZ016072	MH055934	
71	*L.fritinniens*	Danum Valley Field Center, Sabah, Malaysia	FMNH 244800	MH055971	
72	*L.fuliginosa*	Phetchaburi, Thailand	KUHE:20197	LC201988	
73	*L.gracilis*	Bukit Kana, Sarawak, Malaysia	FMNH 273682	MH055972	
74	*L.guinanensis*	Shangsi County, Guangxi, China*	NNU00557	OP548561	
75	*L.hamidi*	Borneo, Malaysia*	KUHE 17545	AB969286	
76	*L.heteropus*	Peninsular, Malaysia	KUHE 15487	AB530453	
77	*L.isos*	Gia Lai, Vietnam*	AMS R 176480	KT824769	
78	*L.itiokai*	Gunung Mulu National Park, Sarawak, Malaysia*	KUHE:55897	LC137805	
79	*L.jinshaensis*	Lengshuihe Nature Reserve, Jinsha County, Guizhou, China*	CIBJS20200516001	MT814014	
80	*L.jinyunensis*	Mt. Jinyun, Beibei District, Chongqing, China*	CIB 119039	OQ024778	
81	*L.juliandringi*	Sarawak, Borneo, Malaysia*	KUHE 17557	LC056784	
82	*L.kajangensis*	Tioman, Malaysia*	LSUHC:4439	LC202002	
83	*L.kalonensis*	Binh Thuan, Vietnam*	IEBR A.2014.15	KR018114	
84	*L.kecil*	Cameron, Malaysia *	KUHE:52439	LC202003	
85	*L.khasiorum*	Meghalaya, India*	SDBDU 2009.329	KY022303	
86	*L.korifi*	Doi Inthanon, Thailand*	KUHE 19134	LC741033	
87	*L.laui*	Wutongshan, Shenzhen city, China*	SYS a001507	KM014544	
88	*L.macrops*	Dak Lak, Vietnam*	AMS R177663	KR018118	
89	*L.maculosa*	Ninh Thuan, Vietnam*	AMS: R 177660	KR018119	
90	*L.maoershanensis*	Mao’er Shan, Guangxi, China	KIZ07614	MH055927	
91	*L.marmorata*	Borneo, Malaysia*	KUHE 53227	AB969289	
92	*L.maura*	Borneo, Malaysia	SP 21450	AB847559	
93	*L.melanoleuca*	Kapoe, Ranong, Thailand	KIZ018031	MH055967	
94	*L.melica*	Ratanakiri, Cambodia*	MVZ 258198	HM133600	
95	*L.minima*	Doi Phu Fa, Nan, Thailand	KIZ024317	MH055852	
96	*L.mjobergi*	Sarawak, Borneo, Malaysia*	KUHE 47872	LC056787	
97	*L.nahangensis*	Tuyen Quang, Vietnam*	ROM 7035	MH055853	
98	*L.namdongensis*	Thanh Hoa, Vietnam*	VNUF A.2017.95	MK965390	
99	*L.neangi*	Veal Veng District, Pursat, Cambodia*	CBC 1609	MT644612	
100	*L.niveimontis*	Yongde County, Yunnan, China *	KIZ028276	MT302620	
101	*L.nyx*	Ha GiangProv., Vietnam*	AMNH A 163810	DQ283381	
102	*L.oshanensis*	Emei Shan, Sichuan, China*	Tissue ID: YPX37492	MH055896	
103	*L.pallida*	Lam Dong, Vietnam*	UNS00510	KR018112	
104	*L.parva*	Mulu National Park, Sarawak, Malaysia*	KUHE:55308	LC056791	
105	*L.pelodytoides*	NA	TZ819	AF285192	
106	*L.petrops*	Ba Vi National Park, Ha Tay, Vietnam	ROM 13483	MH055901	
107	*L.phiadenensis*	Phia Oac-Phia Den NP, Cao Bang Prov., Vietnam*	IEBR A.5205	OR405872	
108	*L.phiaoacensis*	Phia Oac-Phia Den NP, Cao Bang Prov., Vietnam*	IEBR A. 5195	OR405871	
109	*L.picta*	Borneo, Malaysia	UNIMAS 8705	KJ831295	
110	*L.pluvialis*	Lao Cai, Vietnam*	MNHN:1999.5675	JN848391	
111	*L.puhoatensis*	Nghe An, Vietnam*	VNMN 2016 A.22	KY849586	
112	*L.purpurus*	Yunnan, China *	SYSa006530	MG520354	
113	*L.purpuraventra*	Guizhou, China *	SYSa007281	MK414517	
114	*L.pyrrhops*	Loc Bac, Lam Dong, Vietnam*	ZMMU-A-4873-00158	MH055950	
115	*L.rowleyae*	Da Nang City, Vietnam*	ITBCZ2783	MG682552	
116	*L.sabahmontanus*	Borneo, Malaysia*	BORNEENSIS 12632	AB847551	
117	*L.shangsiensis*	Shangsi County, China*	NHMG1401032	MK095460	
118	*L.shimentaina*	Shimentai Nature Reserve, Guangdong, China*	SYS a004712	MH055926	
119	*L.shiwandashanensis*	Shangsi, Guangxi, China*	NNU202103261	MZ326695	
120	*L.sinorensis*	Mae Hong Son, Thailand*	KUHE 19816	LC741036	
121	*L.sola*	Gunung Stong, Kelantan, Malaysia	KU RMB20973	MH055973	
122	*L.suiyangensis*	Guizhou, China *	GZNU20180606005	MK829649	
123	*L.sungi*	Vinh Phuc, Vietnam *	ROM 20236	MH055858	
124	*L.tadungensis*	Dak Nong, Vietnam*	UNS00515	KR018121	
125	*L.tengchongensis*	Yunnan, China *	SYSa004598	KU589209	
126	*L.tuberosa*	Kon Ka Kinh National Park, Gia Lai, Vietnam*	ZMMU-NAP-02275	MH055959	
127	*L.ventripunctata*	Zhushihe, Yunnan, China *	SYSa004536	MH055831	
128	*L.wuhuangmontis*	Pubei County, Guangxi, China *	SYS a003486	MH605578	
129	*L.wulingensis*	Hunan, China *	CSUFT194	MT530316	
130	*L.wumingensis*	Wuming County, Guangxi, China*	NNU 01058	OR194551	
131	*L.yeae*	Mount Emei, Sichuan, China *	CIBEMS20190422HLJ1-6	MT957019	
132	*L.yingjiangensis*	Yunnan, China *	SYSa006532	MG520351	
133	*L.yunkaiensis*	Yunyang County, Chongqing, China *	GZNU20210622001	OL800364	
134	*L.zhangyapingi*	Chiang Mai, Thailand *	KIZ07258	MH055864	
135	*Xenophrysmajor*	Kon Tum Province, Vietnam	AMS R173870	KY476333	
136	*Leptobrachiumchapaense*	Lao Cai Province, Vietnam	AMS R 171623	KR018126	

### ﻿Bioacoustics analyses

Advertisement calls were recorded using a SONY PCM-A10 recorder at a distance of approximately 0.5 m. Four individuals were recorded in the field, and ambient temperatures were measured immediately after the recordings using a Deli LE505 hand-held weather meter. The call recordings were analyzed using the software Raven Pro v. 1.6 (Cornell Laboratory of Ornithology, Ithaca, NY, USA), following the method described by Köhler et al. (2017). Audio-spectrograms were generated by applying a fast-Fourier transform to 512 points with a 50% overlap and a grid-spacing of 172 Hz, using Hanning windows.

## ﻿Results

### ﻿Morphology

**Figure 2.**
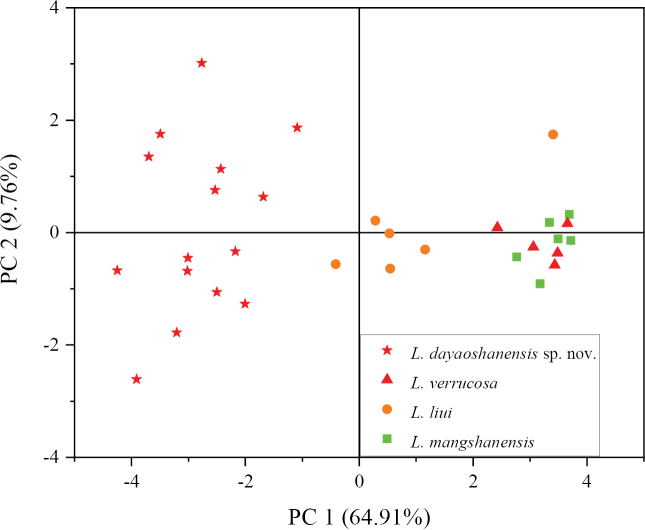
Scatter plot of PC1 and PC2 of PCA based on the morphometric measurements, separating *Leptobrachelladayaoshanensis* sp. nov., *L.liui*, *L.mangshanensis*, and *L.verrucosa*.

The Mann-Whitney *U* test revealed that the DYS specimens differed significantly from *Leptobrachellaliui*, *L.mangshanensis*, and *L.verrucosa* (Table 2) in terms of various morphological characters, such as SVL, HL, HW, SNT, TD, IN, TIB, FLL, ML, and HLL. This finding was further supported by the results of the PCA analysis, which demonstrated that the DYS specimens were distinct from *L.liui*, *L.mangshanensis*, and *L.verrucosa* (Fig. 2). In terms of morphology, the DYS specimens exhibited differences compared to *L.liui* in body size, ventral surface textures, and dermal ridges of toes. Additionally, they differed from *L.mangshanensis* in dorsal surface, lateral fringes of toes, and iris color, and from *L.verrucosa* in lateral fringes of toes, relative finger lengths, and ventrolateral glandular line. For more detailed comparisons, please refer to the “Comparisons” section.

**Table 2.** Measurements and morphometric comparisons between *Leptobrachelladayaoshanensis* sp. nov., *L.liui*, *L.mangshanensis*, and *L.verrucosa*. “*” stands for *p*-value < 0.05.

	***L.dayaoshanensis* sp. nov.**			***p*-value from Mann-Whitney *U* test**		
	**Males (*n* = 15) Ranges (mm)**	**Mean ± SD (mm)**	**Female (*n* = 1)**	**New species vs *L.liui***	**New species vs *L.mangshanensis***	**New species vs *L.verrucosa***
SVL	26.6–28.9	27.9 ± 0.7	34.4	0.000*	0.000*	0.001*
HL	7.8–9.1	8.4 ± 0.3	9.8	0.000*	0.000*	0.001*
HW	9.1–9.9	9.5 ± 0.2	10.9	0.000*	0.000*	0.001*
SNT	3.3–4.1	3.7 ± 0.2	4.1	0.001*	0.001*	0.026*
ED	3.0–4.0	3.5 ± 0.3	4.1	0.020*	0.020*	0.010*
IOD	2.6–3.3	2.9 ± 0.2	3.1	1.000	1.000	0.965
TD	1.3–1.8	1.5 ± 0.1	2.0	0.000*	0.000*	0.002*
TED	0.9–1.4	1.1 ± 0.1	1.4	0.010*	0.010*	0.032*
IN	2.4–2.9	2.6 ± 0.1	2.8	0.000*	0.000*	0.001*
TIB	11.7–13.7	12.9 ± 0.5	15.0	0.001*	0.001*	0.005*
FLL	11.4–13.1	12.4 ± 0.5	13.5	0.000*	0.000*	0.001*
TFL	16.8–19.4	17.8 ± 0.6	20.6	0.003*	0.001*	unknown
ML	6.0–6.9	6.6 ± 0.3	7.1	0.000*	0.000*	0.001*
HLL	36.7–41.2	38.9 ± 1.0	46.2	0.000*	0.000*	0.001*
FG-knee	4.1–5.6	5.0 ± 0.4	5.9	0.000*	0.001*	unknown

### ﻿Phylogenetic analyses and genetic divergence

**Figure 3.**
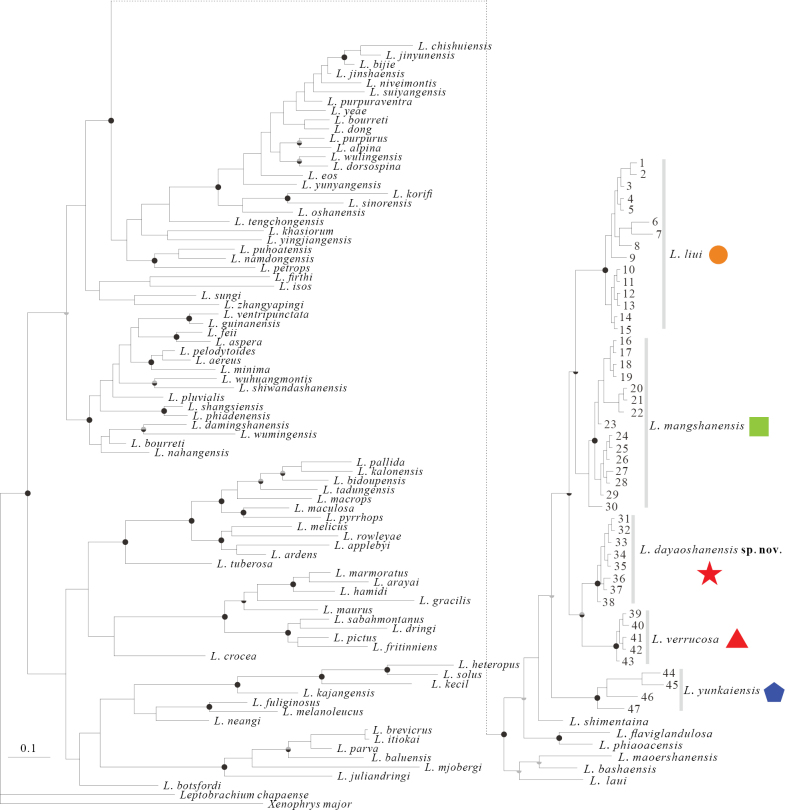
BI trees based on 16S fragments with Bayesian posterior probabilities/bootstrap supports on branches. Bayesian posterior probabilities, upper half, >0.95 = grey, 1 = black; bootstrap supports, lower half, >70%<90% = grey, >90% = black).

About 530 base pairs were included in the matrix of 136 sequences based on the 16S gene. The phylogenetic relationships within *Leptobrachella* remain unresolved in the 16S gene trees. Both ML and BI analyses resulted in similar topological trees (Fig. 3). The phylogenetic trees suggest that the DYS specimens form a monophyletic group and are genetically closest to *L.verrucosa*. Additionally, *L.liui* and *L.mangshanensis* are monophyletic to each other. The genetic divergences between the DYS specimens and *L.verrucosa* range from 2.3% to 2.8%, while for *L.liui* it ranges from 2.6% to 4.1%, and for *L.mangshanensis* it ranges from 2.1% to 2.8% (Suppl. material 2: table S3).

### ﻿Bioacoustics

**Figure 4.**
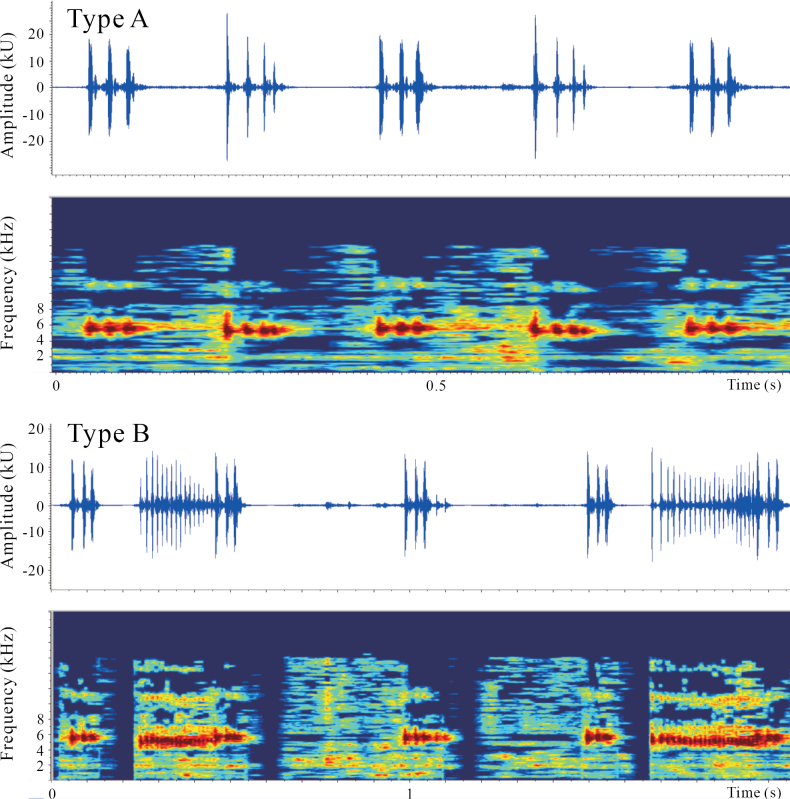
Advertisement call spectrograms of *Leptobrachelladayaoshanensis* sp. nov.

The ambient temperature was recorded at approximately 21.0 °C. The calls of DYS specimens consist of two types: Type A and Type B, as illustrated in Fig. 4. Type A calls have durations ranging from 68 to 88 ms (mean ± SD: 78.1 ± 4.4 ms, *n* = 58) and intervals of 110 to 170 ms (134.6 ± 13.7 ms, *n* = 58), with dominant frequencies between 4.2 and 6.8 kHz. In contrast, Type B calls have durations ranging from 261 to 361 ms (298.5 ± 25.4 ms, *n* = 14) and dominant frequencies also ranging from 4.5 to 6.8 kHz (Fig. 4). These call characters, including durations, intervals, number of notes per call, and dominant frequencies, differentiate the calls of DYS specimens from those of *L.liui* (Ding et al. 2019). Moreover, the calls of DYS specimens also exhibit differences from those of other known species within the *Leptobrachella* genus (Suppl. material 2: table S4). Based on the results of phylogenetic analysis, morphology, and bioacoustics, the DYS specimens are described as a new species within the genus *Leptobrachella*.

### ﻿Taxonomic account

#### ﻿ Leptobrachella dayaoshanensis

Taxon classificationAnimaliaAnuraMegophryidae

Chen, Yu, Meng & Qin sp. nov.

7C067D5F-F5C5-548D-9454-22C5F6E09FE2

https://zoobank.org/FB15638D-4519-4DDA-A95A-007DAEDCEBB7

Fig. 5A–E

Leptolalax liui Fei & Ye, 1990 (Chresonymy): Fei et al. 1992; Fei et al. 2012; Mo et al. 2014.

##### Type material.

***Holotype*** • NNU 20210318, adult male, collected at the Dayaoshan National Nature Reserve (DYS), Jinxiu County, Guangxi, China (24.153°N, 110.213°E; elevation 1132 m), collected by Wei-Cai Chen on 12 March 2021. ***Paratypes*** • NNU 20210319–26, NNU 20210328–33, 14 adult males, and NNU 20210327, one adult female, collected at the same locality as holotype on 12 March 2021 by Wei-Cai Chen.

##### Etymology.

The species name *dayaoshanensis* refers to the type locality, Mount Dayaoshan. The suggested English name is Dayaoshan Leaf Litter Toad, and the Chinese name is Da Yao Shan Zhang Tu Chan (大瑶山掌突蟾).

##### Diagnosis.

*Leptobrachelladayaoshanensis* sp. nov. can be distinguished from its congeners by a combination of the following characters: (1) medium size (SVL mean 27.9 ± 0.7 mm, range 26.6–28.9 mm in males; 34.4 mm in female); (2) dorsal surface rough with small, raised tubercles and ridges; (3) flanks with irregular black spots and creamy-white glands; (4) ventral surface creamy-white with sparse, light-brown spots and irregular, tiny textures; (5) brown throat and chest; (6) rudimentary toe webbing; (7) wide toes lateral fringes; (8) distinct continued ventrolateral glandular line; (9) tibiotarsal articulation reaching the midpoint of eye when the leg is pressed forward; (10) heels not meeting when thighs are appressed at right angles to body; (11) bicolored iris, with upper half copper, gradually transitioning to silver in lower half; and (12) advertisement calls consisting of two types models, with dominant frequencies of 4.2–6.8 kHz (21.0 °C).

##### Description of holotype.

**Figure 5.**
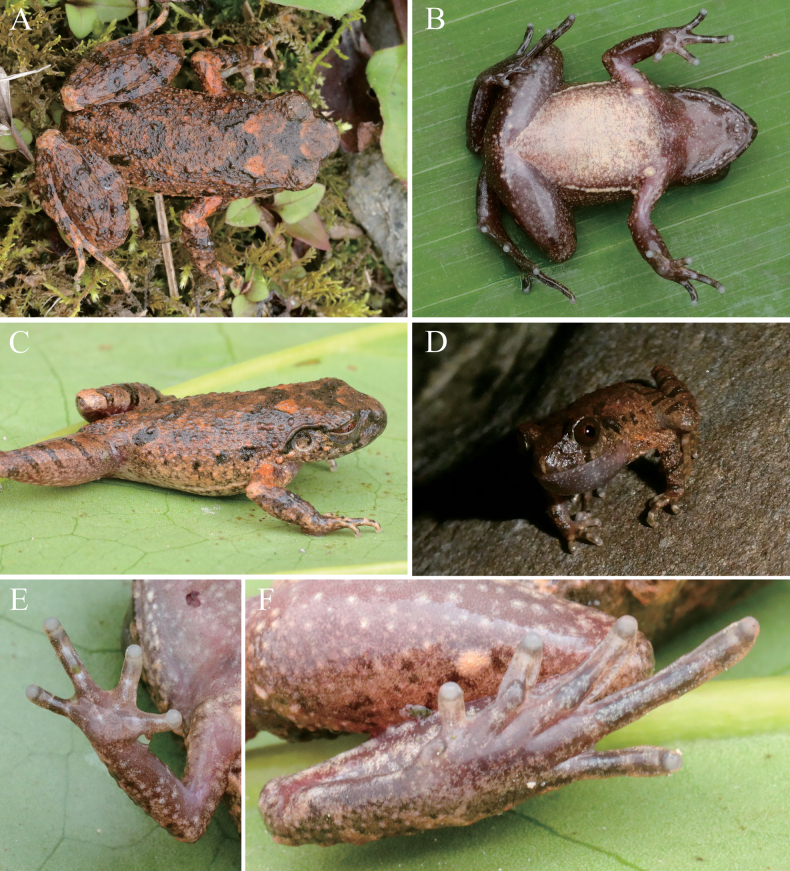
The holotype of *Leptobrachelladayaoshanensis* sp. nov. **A** dorsal view **B** ventral view **C** dorsolateral view **D** calling in the field **E** ventral view of hand **F** ventral view of foot.

Adult male, head width larger than length (HW/HL = 1.08); snout protruding, projecting over the lower jaw; nostril oval, closer to tip of snout than eye; canthus rostralis rounded; loreal region sloping; interorbital area flat; pupil vertical; eye diameter less snout length (ED/SNT = 0.75); internarial distance less than interorbital distance (IN/IOD = 0.77); tympanum distinct, rounded and concave, significantly less than eye diameter, TD/ED = 0.43; distinct and raised supratympanic fold from the corner of eye to supra-axillary gland; vomerine teeth absent; tongue with a shallow notch at the posterior tip (Fig. 5A–F).

Tips of fingers slightly swollen; relative finger lengths I < II < IV < III; nuptial pad absent; subarticular tubercles absent; prominent inner palmar tubercle and very small outer palmar tubercle; finger webbing and dermal fringes absent. Tips of toes rounded, slightly swollen but less than toe width; relative toe lengths I < II < V < III < IV; subarticular tubercles absent, instead by consecutive dermal ridges; large and elongated inner metatarsal tubercle; outer metatarsal tubercle absent; toe webbing rudimentary; toes lateral fringes well-developed, half of phalange in width; TIB/SVL = 0.42; tibia–tarsal articulation reaching the medium of eye when the leg is pressed forward; heels not meeting when thighs are appressed at right angles to body.

Dorsal surface rough with small, raised tubercles and ridges; ventral surface smooth without tubercles; pectoral glands oval and creamy white, ~1.2 mm in diameter; femoral glands oval, ~1.3 mm in diameter, closer to knee than to vent; supra-axillary glands distinct and rounded, ~1.0 mm in diameter; continued ventrolateral glandular line distinct; limbs surface without tubercles (Fig. 5A–F).

##### Color of holotype in life.

Dorsum saffron yellow with distinct brown markings, a brown triangle between eyes, a brown “W” marking on shoulder, and a brown “Λ” marking on lower back; tympanum pale brown; supratympanic line brown from posterior corner of eye to supra-axillary glands; brown bars on upper lip wide; flanks with irregular black spots and creamy yellow glands; three transverse, dark-brown bars distinct on dorsal surface of hindlimbs; elbows, upper arms and tibiotarsal surfaces light tangerine; ventral surface creamy white, with sparse, light-brown spots and irregular, tiny textures; throat and chest brown; pectoral and femoral glands creamy white, supra-axillary glands light tangerine; pupil black; iris bicolored, upper half copper, gradually transitioning to silver in lower half (Fig. 5A–F).

##### Color of holotype in preservative.

Dorsum and limbs surfaces faded to uniform grey; brown triangular marking distinctly visible between eyes; irregular black spots on flanks distinct; throat, chest grey, and belly creamy white; pectoral, femoral, supra-axillary, and ventrolateral glands creamy white; dark bars on limbs, fingers, and toes distinct; elbow pale orange; upper arm and tibiotarsus faded to grey.

##### Variation.

Measurements of the type series are presented in Table 2. The presence of tubercles or ridges on the dorsum varies, as do the irregular markings (Suppl. material 1: fig. S1). Certain specimens exhibit a lighter mustard color. Additionally, the margin of the throat displays creamy-white tubercles in some individuals (Suppl. material 1: fig. S2).

##### Measurements of holotype

**(in mm).**SVL 27.6, HL 8.6, HW 9.3, SNT 4.0, ED 3.0, IOD 3.1, TD 1.3, TED 1.2, IN 2.4, TIB 11.7, FLL 12.2, THL 17.8, ML 6.7, HLL 39.4, FG-knee 4.7.

##### Ecology and distribution.

*Leptobrachelladayaoshanensis* sp. nov. was discovered in the evergreen forest at Mount Dayaoshan, at elevations between 1,000 and 1,600 m. Our observations revealed that adult males of this species were found calling near rocky streams between 19:00 and 24:00 h during our survey. Interestingly, there were also instances where advertisement calls could be heard during the daytime. We noted that the advertisement calls were audible from early March until the end of April. Presently, *L.dayaoshanensis* sp. nov. is only found within the Dayaoshan National Nature Reserve.

##### Comparisons.

*Leptobrachelladayaoshanensis* sp. nov. can be distinguished from other *Leptobrachella* species by its body size (males: SVL 26.6–28.9 mm; female: 34.4 mm); small, raised tubercles and ridges on dorsum; presence of irregular black spots on flanks; creamy-yellow ventral surface with sparse, light-brown spots and irregular textures; rudimentary toe webbing; wide lateral fringes; tongue with a shallow notch; brown throat and chest; distinct continued ventrolateral glandular line; tibiotarsal articulation reaching the midpoint of eye; heels not meeting when thighs are appressed at right angles to body; and bicolored iris, with the upper half being copper, gradually transitioning to silver in the lower half. Furthermore, the species can be identified by its unique advertisement calls (Fig. 4, Suppl. material 2: table S4).

Phylogenetically, *L.dayaoshanensis* sp. nov. is closely related to *L.liui*, *L.mangshanensis*, and *L.verrucosa* (Fig. 3). Morphologically, *L.dayaoshanensis* sp. nov. differs from *L.liui* in having a relatively larger body size in female (SVL 34.4 mm vs SVL 23.1–28.1 mm); HL/HW = 0.92 (vs HL/HW = 1.01); ventral surface creamy yellow, with sparse, light-brown spots and irregular textures, brown throat and chest with scattered, light, creamy spots (vs chest and margins creamy white with dark-brown spots); heels not meeting when thighs are appressed at right angles to body (vs overlapped); consecutive dermal ridges under toes (vs discrete dermal ridges); different call durations (68–88 ms vs 26–78 ms) and dominant frequencies (4.2–6.8 kHz vs 4.8–5.5 kHz). *Leptobrachelladayaoshanensis* sp. nov. differs from *L.mangshanensis* in having a rough dorsal surface with small, raised tubercles and ridges (vs dorsal skin smooth with small orange tubercles and irregular, dark-brown stripes); wide toe lateral fringes (vs narrow toe lateral fringes); distinct longitudinal ridges under toes (vs indistinct longitudinal ridges under toes); tibiotarsal articulation reaching the medium of eye (vs tibiotarsal articulation reaching anterior margin of snout); brown throat and chest, and creamy-white belly with sparse, light-brown spots and irregular, tiny textures (vs greyish-white throat and belly with little white speckles); bicolored iris, upper half copper, gradually transitioning to silver in lower half (vs bright-orange upper, greyish cream below). *Leptobrachelladayaoshanensis* sp. nov. differs from *L.verrucosa* in having relatively larger body size in males (SVL 26.6–28.9 mm vs SVL 23.2–25.9 mm); head width larger than length (HW/HL = 1.08) (vs head length slightly larger than head width, HW/HL = 0.95); eye diameter less snout length (SNT/ED = 1.33) (vs SNT/ED = 1.03); tongue with a shallow notch at the posterior tip (vs tongue deeply notched distally); tibiatarsal articulation reaching the medium of eye when the leg is pressed forward (vs tibiotarsal articulation reaches to anterior corner of eye); rough dorsal surface with small, raised tubercles and ridges (vs shagreened dorsal surface with numerous conical tubercles, lacking spines, enlarged warts, or skin ridges); brown supratympanic line (vs black supratympanic line); wide toe lateral fringes (vs narrow toe lateral fringes); brown throat and chest (vs creamy white); relative finger lengths I < II < IV < III (vs I = II = IV < III); bicolored iris, upper half copper, gradually transitioning to silver in lower half (vs upper half coppery orange, lower half greyish brown); distinct continued ventrolateral glandular line (vs discrete ventrolateral gland line). Furthermore, *L.dayaoshanensis* sp. nov. is found at elevations over 1,000 m, whereas *L.verrucosa* inhabits elevations of 500–600 m. The advertisement calls of the new species can be heard from early March to the end of April, while the breeding season of *L.verrucosa* lasts from April to June according to Lin et al. (2022).

*Leptobrachelladayaoshanensis* sp. nov. differs from *L.shimentaina* and *L.yunkaiensis* in lacking lateral fringes on fingers (vs presence of lateral fringes on fingers), heels not meeting when thighs are appressed at right angles to body (vs overlapped); from *L.flaviglandulosa* in having heels not meeting when adpressed (vs overlapped), consecutive dermal ridges under toes (vs discrete dermal ridges); from *L.bashaensis* in having relatively larger body size (SVL 26.6–28.9 mm in males, 34.4 mm in female vs SVL 22.9–25.6 mm in males, 27.1 mm in female); wide toe lateral fringes (vs narrow toe lateral fringes); from *L.maoershanensis* in having wide toe lateral fringes (vs narrow toe lateral fringes), distinct consecutive dermal ridges under toes (vs indistinct longitudinal ridges under toes); from *L.laui* in lacking finger lateral fringes (vs presence of moderate lateral fringes), creamy-yellow ventral surface with sparse, light-brown spots and irregular textures, brown throat and chest with scattered, light-creamy spots (vs near immaculate creamy-white chest and belly); from *L.phiaoacensis* in having wide toe lateral fringes (vs narrow toe lateral fringes), heels not meeting when thighs are appressed at right angles to body (vs overlapped), continued ventrolateral glandular line (vs discrete ventrolateral gland line), brown throat and chest (vs creamy white).

In having supra-axillary and ventrolateral glands, *L.dayaoshanensis* sp. nov. differs from its congeners from South of the Isthmus of Kra, *L.arayai*, *L.dringi*, *L.fritinniens*, *L.gracilis*, *L.hamidi*, *L.heteropus*, *L.kajangensis*, *L.kecil*, *L.marmorata*, *L.maura*, *L.melanoleuca*, *L.picta*, *L.platycephala*, *L.sabahmontana*, and *L.sola* (vs absent in the latter species). In having a relatively larger body size (SVL 26.6–28.9 mm in males, 34.4 mm in females), *L.dayaoshanensis* sp. nov. differs from *L.baluensis* (SVL 14.9–15.9 mm in males), *L.bondangensis* (SVL 17.8 mm in male), *L.brevicrus* (SVL 17.1–17.8 mm in males), *L.fusca* (SVL 16.3 mm in male), *L.itiokai* (SVL 15.2–16.7 mm in males), *L.juliandringi* (SVL 17.0–17.2 mm in males), *L.mjobergi* (SVL 15.7–19.0 mm in males), *L.natunae* (SVL 17.6 mm in male), *L.palmata* (SVL 14.4–16.8 mm in males), *L.parva* (SVL 15.0–16.9 mm in males), and *L.serasanae* (SVL 16.9 mm in female).

For the remaining known *Leptobrachella* species from north of the Isthmus of Kra, in having SVL 26.6–28.9 mm in males, *L.dayaoshanensis* sp. nov. differs from the smaller *L.applebyi* (19.6–22.3 mm), *L.ardens* (21.3–24.7 mm), *L.bidoupensis* (18.5–25.4 mm), *L.melica* (19.5–22.7 mm), *L.niveimontis* (22.5–23.6 mm), *L.pluvialis* (21.3–22.3 mm), *L.rowleyae* (23.4–25.4 mm); from the larger *L.dushanensis* (31.9–32.9 mm), *L.nahangensis* (40.8 mm), *L.sungi* (48.3–52.7 mm), and *L.zhangyapingi* (45.8–52.5 mm).

Furthermore, in having wide lateral fringes on toes, *L.dayaoshanensis* sp. nov. differs from *L.applebyi*, *L.ardens*, *L.crocea*, *L.kalonensis*, *L.lateralis*, *L.macrops*, *L.maculosa*, *L.melica*, *L.nahangensis*, *L.namdongensis*, *L.neangi*, *L.nyx*, *L.oshanensis*, *L.pallida*, *L.pluvialis*, *L.pyrrhops*, *L.rowleyae*, *L.shiwandashanensis*, *L.tadungensis*, *L.tuberosa* and *L.ventripunctatus* (vs absent lateral fringes on toes). In having black spots on flanks, *L.dayaoshanensis* sp. nov. differs from *L.aerea*, *L.botsfordi*, *L.crocea*, *L.eos*, *L.firthi*, *L.graminicola*, *L.isos*, *L.pallida*, *L.petrops* and *L.tuberosa* (vs absent).

*Leptobrachelladayaoshanensis* sp. nov. differs from its congeners in the dominant frequency of 4.2–6.8 kHz (21.0 °C) (Suppl. material 2: table S4).

## ﻿Discussion

Preprimary phylogenetic trees revealed that sequences named *Leptobrachellaliui* downloaded from GenBank (Chen et al. 2018; Frost 2024) did not form a monophyletic group but could be divided into four lineages. Among these lineages, the DYS specimens form a monophyletic group closely related to *L.verrucosa*. Despite the close phylogenetic relationship, the DYS specimens and *L.verrucosa* are distinct, as confirmed by PCA, Man-Whitney *U* test, and morphological characters (see comparison section). Therefore, the DYS specimens can be classified as a new species of *Leptobrachella*. Three sequences named *L.liui* from southwestern Guangdong were clustered with *L.yunkaiensis* (paratype) with well-supported values (Fig. 3). Due to their close geographical distance and low genetic divergence (Suppl. material 2: table S3), we assigned these three sequences to *L.yunkaiensis*. Sequences named *L.liui* but nested within *L.mangshanensis* were treated as *L.mangshanensis*, which are found in the borders between Hunan and Jiangxi, and between Guangxi, Guangdong, and Hunan (Fig. 1). The sensu stricto *L.liui* occurs in and around the type locality (Wuyishan, Fujian, China), as well as northern and eastern Guangdong (Fig. 1). Further field investigations are required to determine whether *L.liui* and *L.mangshanensis* have sympatric distributions.

Additionally, it should be noted that the taxonomy of the population of Leishan, Guizhou reported by Fei et al. (1992) has not been considered within the *L.liui* species complex. In recent years, several new species of *Leptobrachella* have been discovered in Guizhou (AmphibiaChina 2024). However, there is no evidence supporting the existence of *L.liui* in Guizhou. Therefore, we conclude that *L.liui* does not exist in Guizhou.

## Supplementary Material

XML Treatment for Leptobrachella dayaoshanensis

## ﻿Additional information

### Author ORCIDs

Gui-Dong Yu https://orcid.org/0009-0008-1928-6594

Peng Li https://orcid.org/0000-0001-8311-0544

Wan-Xiao Peng https://orcid.org/0000-0001-5635-9061

Wei-Cai Chen https://orcid.org/0000-0002-2398-4079

### Data availability

All of the data that support the findings of this study are available in the main text or Supplementary Information.

## Supplementary materials

Supplementary material 1 Supplementary figures Data type pdf Explanation note **figure S1.** Dorsal surface compared between holotype and some paratypes. **A**NNU 20210318, **B**NNU 20210322, **C**NNU 20210324, **D**NNU 20210325, **E**NNU 20210330, **F**NNU 20210327; **figure S2.** Ventral surface compared among some paratypes and ventral view of foot. **A**NNU 20210320, **B**NNU 20210322, **C**NNU 20210324, **D**NNU 20210327, **E**NNU 20210330, **F**NNU 20210332, **G**NNU 20210322, **H**NNU 20210325. https://binary.pensoft.net/file/1184250This dataset is made available under the Open Database License (http://opendatacommons.org/licenses/odbl/1.0/). The Open Database License (ODbL) is a license agreement intended to allow users to freely share, modify, and use this Dataset while maintaining this same freedom for others, provided that the original source and author(s) are credited.Gui-Dong Yu, Kun Qin, Tao Meng, Peng Li, Wan-Xiao Peng, Wei-Cai Chen

Supplementary material 2 Supplementary tables Data type xlsx Explanation note **table S1**: References for morphological characters for congeners of the genus Leptobrachella; **table S2**: Comparative material examined; **table S3**: Uncorrected p-distance in Leptobrachella species based on 16S gene fragments; **table S4**: Dominant frequency of advertisement calls of species available in the genus Leptobrachella. https://binary.pensoft.net/file/1184251This dataset is made available under the Open Database License (http://opendatacommons.org/licenses/odbl/1.0/). The Open Database License (ODbL) is a license agreement intended to allow users to freely share, modify, and use this Dataset while maintaining this same freedom for others, provided that the original source and author(s) are credited.Gui-Dong Yu, Kun Qin, Tao Meng, Peng Li, Wan-Xiao Peng, Wei-Cai Chen
