# Supplementary material for: ﻿A new species of the genus Leptobrachella (Amphibia, Anura, Megophryidae) from Dayaoshan National Nature Reserve, Guangxi, China
Source: Zookeys. 2024 Nov 27;1219:105–22. doi: 10.3897/zookeys.1219.121027 (PMC11618101; doi:10.3897/zookeys.1219.121027)
Supplement: Supplementary material 1 — Supplementary figures [file zookeys-1219-105_article-121027__-s001.pdf]

Suppl. material 1

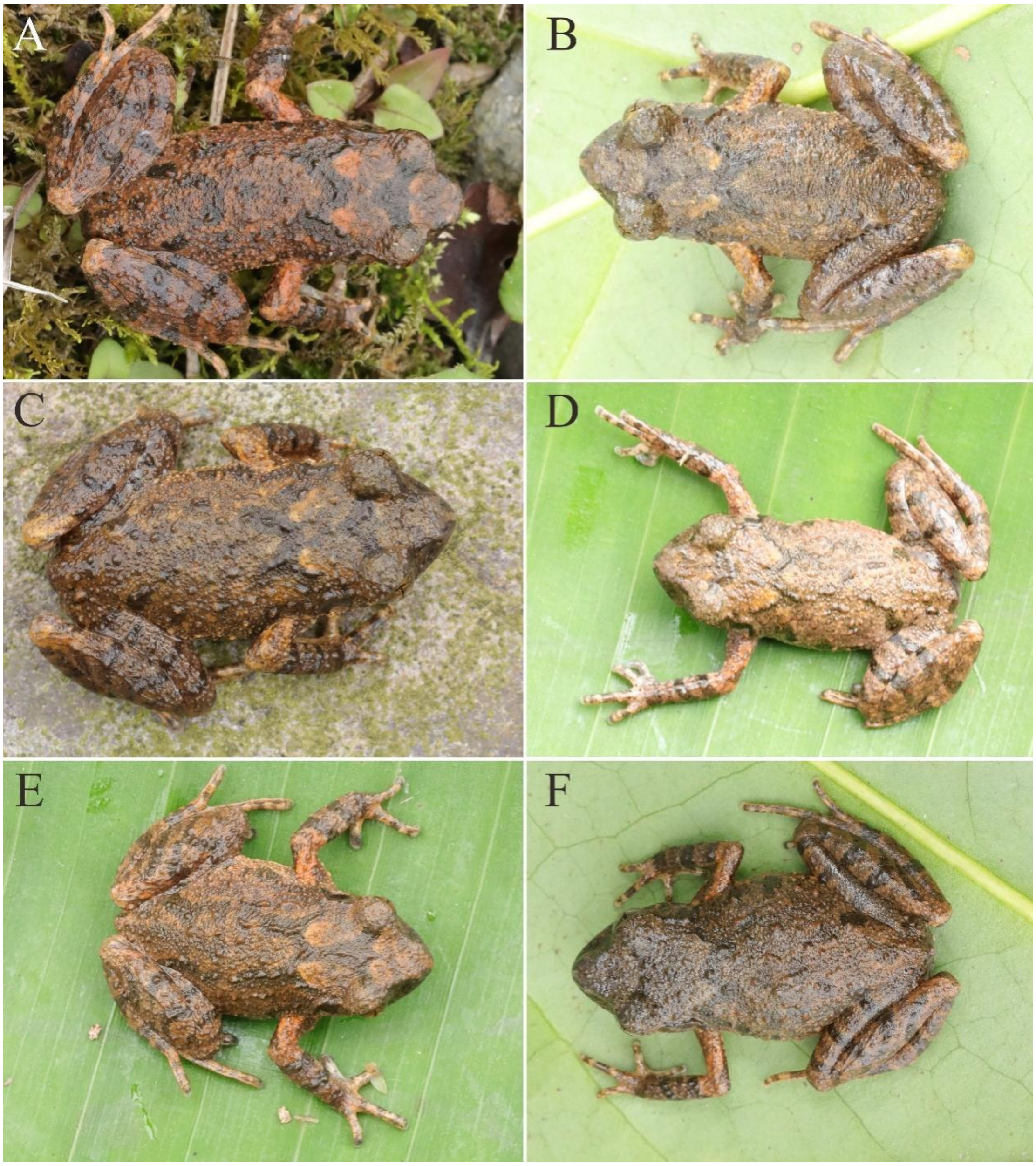

Figure S1. Dorsal surface compared between holotype and some paratypes. **A** NNU 20210318, **B** NNU 20210322, **C** NNU 20210324, **D** NNU 20210325, **E** NNU 20210330, **F** NNU 20210327.

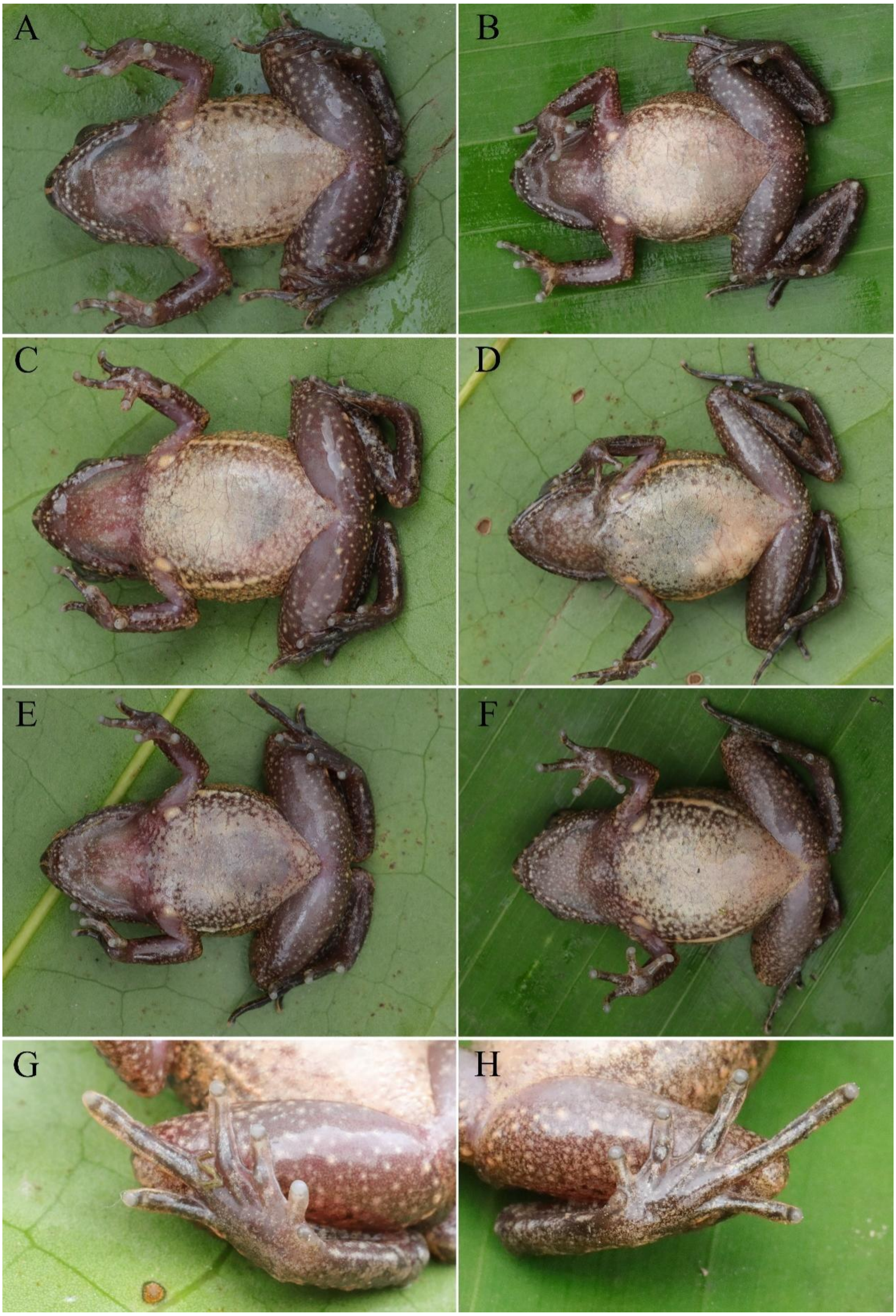

Figure S2. Ventral surface compared among some paratypes and ventral view of foot. **A** NNU 20210320, **B** NNU 20210322, **C** NNU 20210324, **D** NNU 20210327, **E** NNU 20210330, **F** NNU 20210332, **G** NNU 20210322, **H** NNU 20210325.
